# Effect of Tempering Temperature on Hydrogen Embrittlement of SCM440 Tempered Martensitic Steel

**DOI:** 10.3390/ma16165709

**Published:** 2023-08-21

**Authors:** Sang-Gyu Kim, Jae-Yun Kim, Byoungchul Hwang

**Affiliations:** 1 Department of Materials Science and Engineering, Seoul National University of Science and Technology, Seoul 01811, Republic of Korea; rlatkdrb0323@gmail.com (S.-G.K.); sbdowalr@gmail.com (J.-Y.K.); 2 R&D Center, SeAH Besteel, Gunsan 54007, Republic of Korea

**Keywords:** SCM440, hydrogen embrittlement, tempered martensitic steel, tempering temperature

## Abstract

The effect of tempering temperature on the hydrogen embrittlement characteristics of SCM440 tempered martensitic steels was investigated in terms of their microstructure and hydrogen desorption behavior. The microstructures were characterized using scanning and transmission electron microscopy, as well as X-ray diffraction and electron backscattered diffraction analysis. Thermal desorption analysis (TDA) was performed to examine the amount and trapping behavior of hydrogen. The cementite morphology of the SCM440 tempered martensitic steels gradually changed from a long lamellar shape to a segmented short-rod shape with an increasing tempering temperature. A slow strain rate tensile test was conducted after electrochemical hydrogen charging to evaluate the hydrogen embrittlement resistance. The hydrogen embrittlement resistance of the SCM440 tempered martensitic steels increased with an increasing tempering temperature because of the decrease in the fraction of the low-angle grain boundaries and dislocation density. The low-angle grain boundaries and dislocations, which acted as reversible hydrogen trap sites, were critical factors in determining the hydrogen embrittlement resistance, and this was supported by the decreased diffusible hydrogen content as measured by TDA. Fine carbides formed in the steel tempered at a relatively higher temperature acted as irreversible hydrogen trap sites and contributed to improving the hydrogen embrittlement resistance. Our findings can suggest that the tempering temperature of SCM440 tempered martensitic steel plays an important role in determining its hydrogen embrittlement resistance.

## 1. Introduction

Tempered martensitic steels have been widely used in high-pressure hydrogen vessels owing to their excellent combination of strength and ductility [1]. However, hydrogen embrittlement is a critical issue that limits the use of high-strength martensitic steels in high-pressure vessels for hydrogen storage. When steel is exposed to a hydrogen environment, hydrogen atoms can penetrate and interact with all types of microstructural defects, such as lattices, dislocations, grain boundaries, and carbide interfaces, resulting in premature failure of the steel [2,3,4,5,6]. Currently, the accepted hydrogen embrittlement mechanisms include the hydrogen-enhanced decohesion (HEDE) [7], hydrogen-enhanced localized plasticity (HELP) [8], and hydrogen-enhanced strain-induced vacancy (HESIV) mechanisms [9]. The deleterious effects of hydrogen on mechanical properties have motivated the development of metallurgical strategies to improve the hydrogen embrittlement resistance of high-strength martensitic steels.

The susceptibility of steel to hydrogen embrittlement depends on its microstructure [10,11,12]. Tempering is a typical heat treatment process that reduces the risk of hydrogen embrittlement in martensitic steels by decreasing the dislocation density and population of sub-boundaries, thereby leading to improved toughness but lower strength [13]. A series of precipitates is introduced by adjusting the tempering process to compensate for the loss of strength and, in some cases, increase the hydrogen embrittlement resistance [14,15,16]. Zafra et al. [17] reported that the improved hydrogen embrittlement resistance of Cr-Mo low-alloy steels resulted from the lower dislocation density after the tempering process. This indicates that dislocation is a reversible trap site that has a negative effect on the hydrogen embrittlement resistance, and that the dislocation density can be reduced by relieving the internal stress and decomposing martensite during the tempering process. Kang et al. [18] reported that the hydrogen embrittlement resistance was enhanced by increasing the spheroidization rate of fine carbides. The spheroidized carbides inhibited the initiation and propagation of microcracks, whereas the long needle-like carbides facilitated the propagation of microcracks. Moreover, it is reported that nanoscale precipitates in steel, such as Cu-rich precipitates and Cr_2_O_3_ particles with sizes of 10 nm, are beneficial for improving the hydrogen embrittlement resistance [19,20].

This paper reports the effect of tempering temperature on the hydrogen embrittlement resistance of SCM440 tempered martensitic steels. The hydrogen trap state and hydrogen trapping ability were examined using microstructural characterization and thermal desorption analysis. In addition, a slow strain rate test was conducted after electrochemical hydrogen charging to investigate the tensile properties and fracture behavior.

## 2. Materials and Methods

The chemical composition of the SCM440 steels investigated in this study was Fe-0.40C-0.25Si-0.68Mn-1.04Cr-0.19Mo (wt.%). These steels were austenitized at 880 °C for 30 min, followed by oil cooling, tempering at 300, 450, and 600 °C for 60 min, and water cooling. After the longitudinal–transverse planes of the steels were polished and etched with a 3% nital solution, the microstructures were observed using field-emission scanning electron microscopy (FE-SEM, Inspect F, FEI, USA). Electron backscatter diffraction (EBSD) analysis was also performed to observe the microstructures and the orientation imaging software provided by TSL (TexSEM Laboratories, Inc., Draper, UT, USA) was used for analyzing the EBSD data. Carbide precipitation was confirmed using X-ray diffraction (XRD, Bruker DE/D8 Advance, Bruker, Germany). Each specimen was subjected to electrolytic extraction, and XRD analysis was performed to investigate the carbide precipitation behavior. Corrected scanning transmission electron microscopy (Cs-TEM, NEO ARM, JEOL, USA) was used for a more detailed microstructural characterization. Transmission electron microscopy (TEM) samples were prepared by carbon extraction replication.

**Figure 1:**
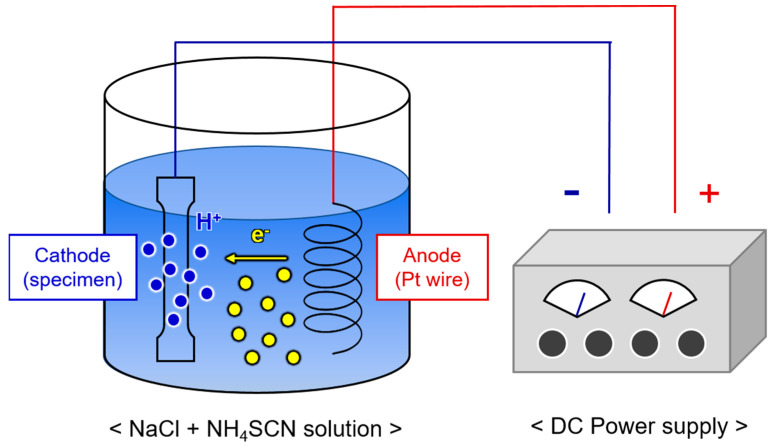
Schematic diagram of the electrochemical hydrogen charging equipment.

A slow strain rate tensile test was performed after electrochemical hydrogen charging to evaluate the hydrogen embrittlement resistance. Subsized plate-type tensile specimens (ASTM E8 standard [21]) were manufactured parallel to the rolling direction. The gauge length, width, and thickness of the tensile specimen were 25.0, 6.3, and 2.0 mm, respectively. The slow strain rate tensile test was conducted at a strain rate of 5.0 × 10^−5^ s^−1^ using a universal testing machine (UT-100E, MTDI, Korea) with a capacity of 10 tons. Some of the tensile specimens were electrochemically hydrogen-charged in an aqueous solution of 3 wt.% NaCl and 0.3 wt.% NH_4_SCN at a current density of 25 A/m^2^ for 24 h using a Pt wire as a counter electrode. A schematic diagram of the electrochemical hydrogen charging equipment is shown in Figure 1. The specimens before and after hydrogen charging were referred to as ‘non-charged’ and ‘H-charged’, respectively. Fractographs of the specimens subjected to the tensile testing before and after hydrogen charging were obtained using FE-SEM. Relative elongation (RE) and relative reduction in area (RRA) were used to quantitatively evaluate the brittleness caused by hydrogen precharging. The RE is the ratio of the longation of the hydrogen-affected specimen to that of the as-received specimen in an ambient environment. The RRA is the ratio of the reduction in the area of the hydrogen-affected specimen to that of the as-received specimen in an ambient environment.

Thermal desorption analysis was performed using a gas chromatography system (7890A, Agilent Technologies, USA) and a tubular furnace under Ar gaseous atmosphere. For the thermal desorption analysis, a rectangular specimen with dimensions of 30 mm × 10 mm × 2 mm was heated up to 600 °C at a rate of 100 °C/h. The total amount of hydrogen desorbed from the investigated steels was calculated by integrating the plot of the desorption rate versus time. The thermal desorption analysis samples were transferred to a tubular furnace within 5 min after hydrogen charging to prevent hydrogen diffusion into the air.

## 3. Results and Discussion

### 3.1. Influence of Tempering Temperature on Microstructure

**Figure 2:**
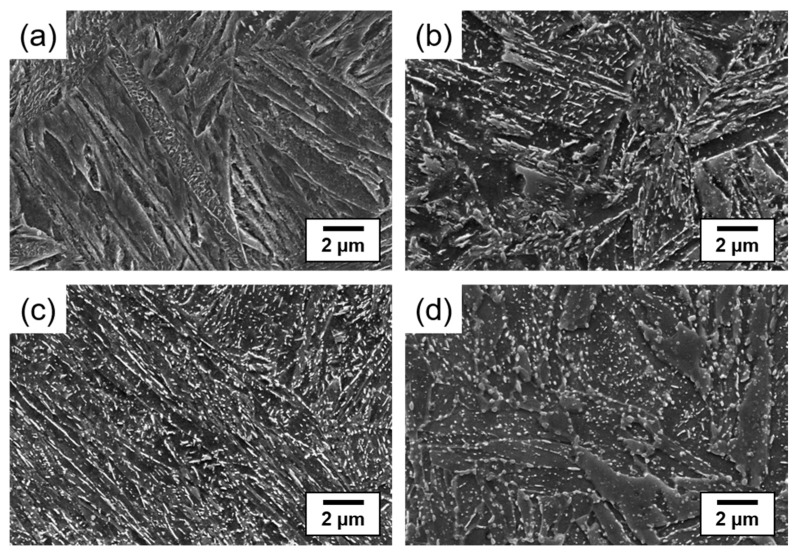
SEM micrographs of the (**a**) as-quenched and (**b**–**d**) tempered SCM440 steels at different tempering temperatures: (**b**) 300 °C, (**c**) 450 °C, and (**d**) 600 °C.

The SEM microstructures of the SCM440 steels tempered at different temperatures are shown in Figure 2. The as-quenched steel exhibited a lath martensitic microstructure owing to rapid cooling after austenitization (Figure 2a). The microstructure of the tempered steel was composed of tempered martensite and cementite precipitated during the tempering process. The cementite in the steels tempered at 300 and 450 °C was long and lamellar (Figure 2b,c), while that in the steel tempered at 600 °C was segmented into short rods (Figure 2d). This morphological difference is due to the gradual spheroidization of the cementite, a process which reduces the surface energy and is influenced by tempering temperature [22].

The dislocation densities of the SCM440 steels tempered at different temperatures were measured using the Williamson–Hall method (Equation (1)) [23]:

(1) $${\mathrm{FWHM}}_{\left(sample\right)}\frac{cos\theta }{\lambda }=\frac{0.9}{D}+2\varepsilon \frac{sin\theta }{\lambda }$$

The full-width half maximum (FWHM) was obtained from the XRD patterns and calculated for different peaks corresponding to the ferrite diffraction planes (*D*), the average grain size, and X-ray wavelength (*λ* = 1.5406 nm). Equation (2) [24,25] was used to determine the dislocation density:

(2) $$\rho =14.4{\left(\frac{\varepsilon }{b}\right)}^{2}$$

Here, b is the Burgers vector (*b* = 0.248 nm) [26] and *ε* can be calculated from the slope of the β∙cosθ versus 4∙sinθ plot.

**Figure 3:**
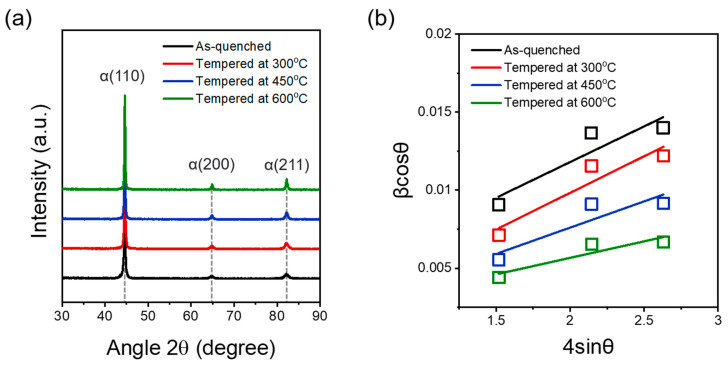
(**a**) XRD patterns of the SCM440 steels tempered at different temperatures. (**b**) Variation in dislocation density as a function of tempering temperature calculated through Williamson–Hall plot method [23].

Figure 3b shows the β∙cos(θ) values for different peaks of each sample. The dislocation densities of the SCM440 steels tempered at different temperatures were obtained from the linear fits and are summarized in Table 1. As shown previously [27], the dislocation density decreases with an increasing tempering temperature. Dislocations can be regarded as reversible hydrogen trap sites, and the accumulation of dislocations in grains causes hydrogen concentration through the trapping effect, thus decreasing the hydrogen embrittlement resistance [28].

**Figure 4:**
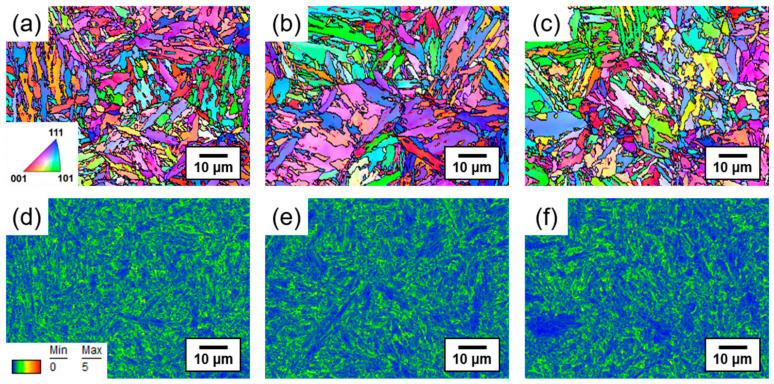
(**a**–**c**) EBSD inverse pole figure (IPF) and grain boundary (GB) maps, and (**d**–**f**) kernel average misorientation (KAM) maps of the SCM440 steels tempered at different temperatures: (**a**,**d**) As-quenched and tempered at (**b**,**e**) 300 °C and (**c**,**f**) 600 °C.

Figure 4 shows the EBSD inverse pole figure (IPF), grain boundary (GB), and kernel average misorientation (KAM) maps of the SCM440 steels tempered at different temperatures. The lath martensitic structure formed by quenching was probably retained in all the steels, although no preferred orientation along the rolling direction was observed (Figure 4a–c). As the tempering temperature increased, the low-angle grain boundary (LAGB) fraction decreased (Table 1). LAGBs are regarded as effective reversible hydrogen trap sites, while the high-angle grain boundary (HAGB) is regarded as an irreversible hydrogen trap site that cannot effectively release hydrogen at room temperature [29]. Another study demonstrated that grain boundaries with low misorientation angles are usually the preferred areas for hydrogen segregation and affect the hydrogen diffusion mechanism [30]. On the other hand, high-angle random boundaries are considered a disordered phase that can serve as a short-circuit path for hydrogen, thereby accelerating the hydrogen diffusion [30,31,32,33,34]. Therefore, the effect of the grain boundary on hydrogen diffusion is two-fold and depends on the competition between these two mechanisms.

The KAM values can be used to determine the geometrically necessary dislocation (GND) density and stress status of the samples [31]. The KAM maps and distributions of the SCM440 steels tempered at different temperatures are shown in Figure 4d–f. Regions with higher KAM values were identified to concentrate near the LAGBs. As grains with higher KAM values are associated with a higher defect density, this observation implied that the steel tempered at 600 °C had a lower GND density than the steel tempered at 300 °C. In the present study, both the GND density and the dislocation density calculated from the XRD analysis tended to decrease with an increasing tempering temperature (Table 1) [35,36,37].

**Figure 5:**
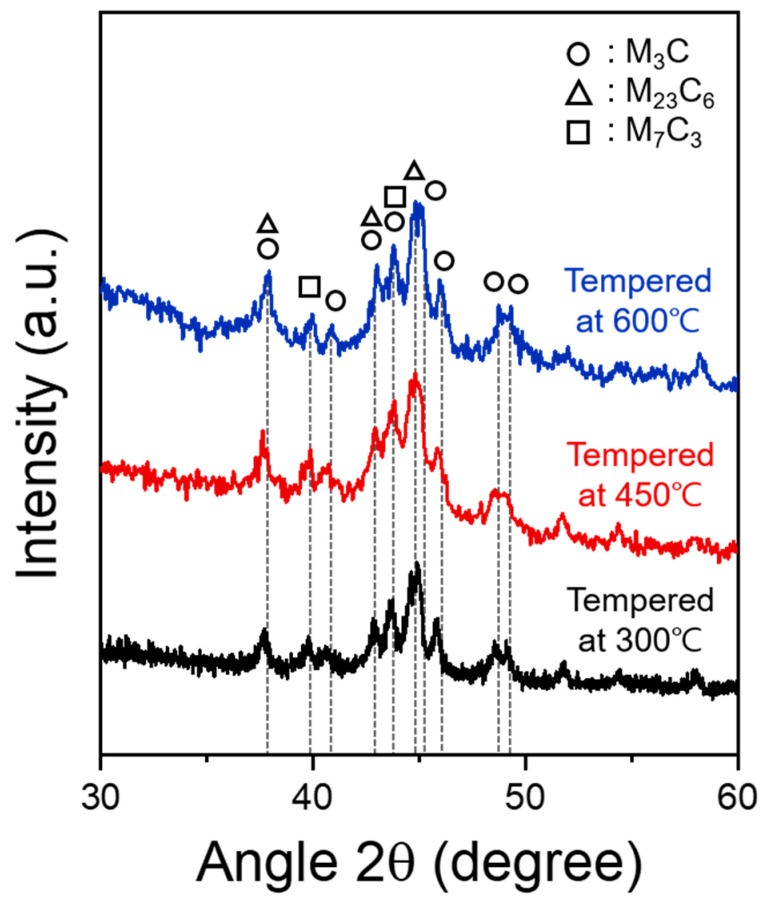
X-ray diffraction patterns of the electrolytic extracted samples for the SCM440 steels tempered at different temperatures.

**Figure 6:**
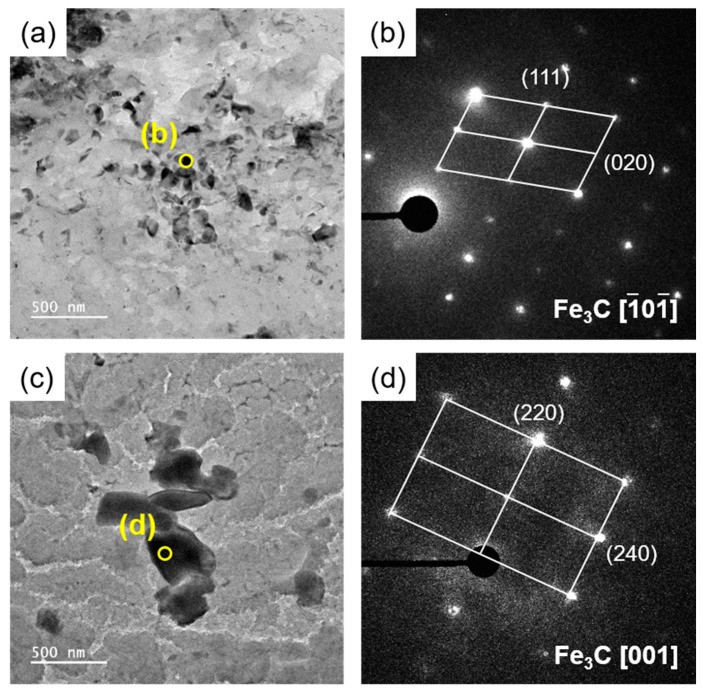
TEM micrographs and selected area electron diffraction (SAED) patterns of carbon extraction replica prepared from the SCM440 steels tempered at (**a**,**b**) 300 °C and (**c**,**d**) 600 °C.

In all the XRD patterns of the electrolytically extracted samples of the SCM440 steels tempered at different temperatures (Figure 5), the main precipitated phases were M_3_C (cementite), M_23_C_6_, and M_7_C_3_ carbide. Among these carbides, the cementite exhibited the most dominant diffraction peak for all the three tempered samples. This suggests that the volume fraction of the cementite was larger than that of the other carbides. It is also important to note that the particles had different sizes, and very fine particles are difficult to analyze using XRD. Fine particles exhibited diffraction broadening, and these peaks were difficult to distinguish from the background. Figure 6 shows the bright-field TEM images of the carbides in the steels tempered at 300 and 600 °C. The selected-area electron diffraction (SAED) pattern showed that the carbides were composed mostly of cementite. The larger fraction of cementite is consistent with the TEM characterization, which indicates substantially lower probabilities of detecting carbides other than cementite.

### 3.2. Influence of Tempering Temperature on Tensile Properties and Hydrogen Embrittlement

**Figure 7:**
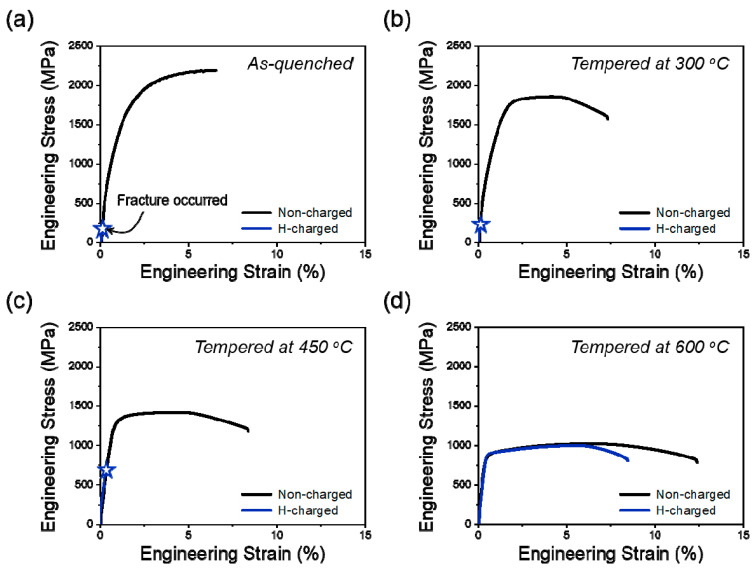
Engineering stress–strain curves of the (**a**) as-quenched and (**b**–**d**) tempered SCM440 steels at different tempering temperatures: (**b**) 300 °C, (**c**) 450 °C, and (**d**) 600 °C. The specimens before electrochemical hydrogen charging were marked as ‘non-charged’, and the specimens after electrochemical hydrogen charging were referred to as ‘H-charged’.

The hydrogen embrittlement resistance of the SCM440 steels tempered at different temperatures was evaluated by comparing their tensile properties. Figure 7 shows the engineering stress–strain curves of the SCM440 steels tempered at different temperatures. The tensile properties, RRA, and RE were calculated from the curves and are summarized in Table 2. The tensile strength decreased and elongation increased with an increase in the tempering temperature. This can be attributed to the softening effect of the tempering process, which reduced the dislocation density. After hydrogen charging, the total elongation and reduction in area decreased dramatically for all the steels. In particular, the as-quenched steel and steels tempered at relatively low temperatures fractured before yielding, whereas the steel tempered at 600 °C fractured after yielding, indicating its higher hydrogen embrittlement resistance. The variation in hydrogen embrittlement resistance is mainly attributed to changes in the dislocation density. Dislocations can be regarded as reversible hydrogen trap sites, and the accumulation of dislocations can cause hydrogen to concentrate, thereby decreasing the hydrogen embrittlement resistance [28].

**Figure 8:**
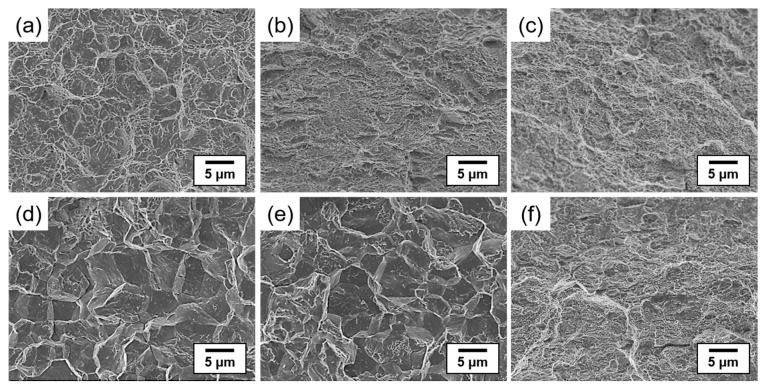
SEM fractographs of (**a**–**c**) non-charged specimen and (**d**–**f**) H-charged specimen for the SCM440 steels tempered at different temperatures: (**a**,**d**) as-quenched and tempered at (**b**,**e**) 300 °C and (**c**,**f**) 600 °C.

Figure 8 shows the fracture morphologies of the as-quenched and tempered (300 and 600 °C) steel specimens under non-charged and H-charged conditions. Before hydrogen charging, all the specimens exhibited a ductile fracture behavior with dimples and micro-void coalescence. On the other hand, intergranular and quasi-cleavage fracture modes coexisted after hydrogen charging in the as-quenched steel and steel specimens tempered at 300 °C. In addition, hydrogen-assisted crack initiation was observed at the grain boundaries, as shown in Figure 8d,e. The mechanism of hydrogen-assisted crack formation was explained based on the combined effect of the HELP [8] and HEDE [7] mechanisms. Hydrogen diffusion occurs through two pathways: dislocation transmission and stress-induced hydrogen enrichment. During tensile testing, hydrogen migration can occur along with dislocations when the dislocation velocity is lower than a critical value [38]. When dislocations move and interact with barriers, hydrogen atoms can be deposited at the grain boundaries owing to weak dislocation traps, resulting in hydrogen accumulation at the boundaries. In addition, dislocation pile-up at the grain boundaries can lead to local work hardening, triggering stress-induced hydrogen diffusion into the boundaries. On the other hand, in the steel specimen tempered at 600 °C, the ductile fracture morphologies were dominant even after hydrogen charging, which correlated well with the result of the slow strain rate tensile test showing relatively high hydrogen embrittlement resistance (Figure 8f).

**Figure 9:**
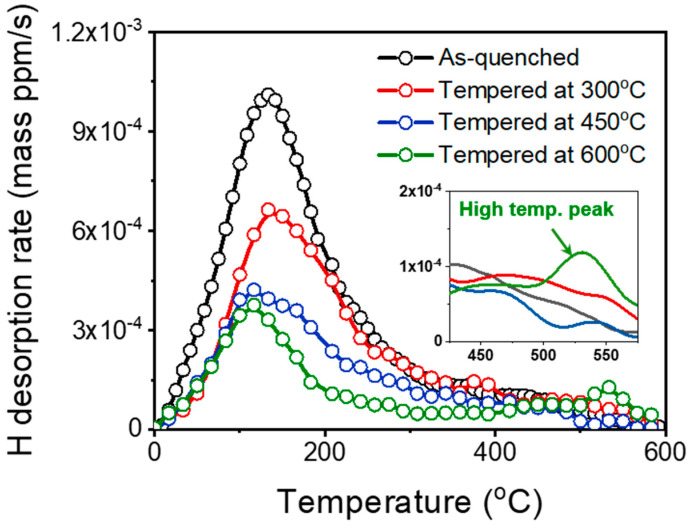
Hydrogen desorption curves at the heating rate 100 °C/h of SCM440 steels tempered at different temperatures. The embedded figure is the locally enlarged view of the desorption curves from 425 °C to 575 °C for each specimen.

Figure 9 shows the results of the thermal desorption analyses of the as-quenched and tempered steel specimens. The hydrogen trapping ability of the steel specimens decreased after the tempering process and also decreased with an increasing tempering temperature. In carbon steels, tempering usually decreases the hydrogen trapping ability of martensitic steels owing to a reduction in the dislocation density [39]. Moreover, cementite has been shown to have a lower hydrogen trapping ability [40]. As shown in the embedded picture in Figure 9, a high-temperature desorption peak around 540 °C was confirmed for the steel tempered at 600 °C. The desorption peaks at higher temperatures correspond to strong trap sites with higher activation energies, and the hydrogen desorbed at high temperatures can be considered irreversible hydrogen. The fine carbides that formed in the steel tempered at 600 °C acted as an irreversible hydrogen trap sites and contributed to improving the hydrogen embrittlement resistance. With an increasing tempering temperature, the increase in fine carbides precipitated within the grains lowered the tendency for intergranular fracture, as also observed by Nagao et al. for low-carbon martensitic plate steels [13].

**Figure 10:**
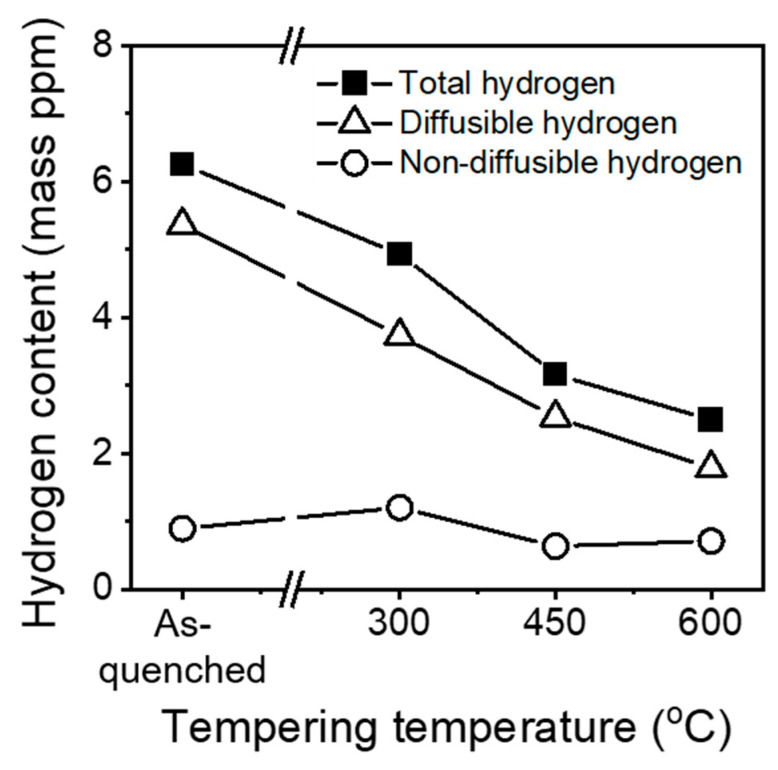
Variation in hydrogen content according to tempering temperatures in the SCM440 steels. Diffusible hydrogen was defined as the hydrogen content desorbed at a temperature below 300 °C, and non-diffusible hydrogen was defined as the hydrogen content desorbed at a temperature between 300 °C and 600 °C.

Figure 10 shows the diffusible and non-diffusible hydrogen contents plotted as a function of the tempering temperature. Diffusible hydrogen was defined as the hydrogen content desorbed at temperatures below 300 °C, while non-diffusible hydrogen was defined as the hydrogen content desorbed at temperatures between 300 and 600 °C [41]. Generally, if steel includes several types of trapping sites that have different activation energies, the hydrogen embrittlement resistance decreases proportionally with the diffusible hydrogen content rather than with the total hydrogen content [42]. In this study, the fraction of diffusible hydrogen decreased with an increasing tempering temperature. These results suggest that the tempering temperature of SCM440 tempered martensitic steel plays an important role in determining its hydrogen embrittlement resistance.

## 4. Conclusions

Based on the present investigation of the effect of tempering temperature on the hydrogen embrittlement of SCM440 tempered martensitic steels, the following conclusions can be drawn:

1. SCM440 steels fabricated by quenching and tempering at various temperatures exhibited a microstructure composed of tempered martensite and cementite, whereas the as-quenched SCM440 steel had a fully lath-like martensitic structure. As the tempering temperature increased, the morphology of cementite changed from a long lamellar shape to a segmented short-rod shape via gradual spheroidization to reduce the surface energy of the cementite.
2. The hydrogen embrittlement resistance of SCM440 tempered martensitic steels increased with an increasing tempering temperature, and this was mainly attributed to the decrease in the fraction of low-angle grain boundaries and dislocation density, which act as reversible hydrogen trap sites.
3. In the as-quenched steel and steel specimen tempered at 300 °C with a relatively high dislocation density after electrochemical hydrogen charging, intergranular and quasi-cleavage fracture modes were observed, and hydrogen-assisted cracks were initiated from the grain boundaries. The mechanism of hydrogen-assisted crack formation can be attributed to the combined effect of the HELP and HEDE mechanisms.
4. Thermal desorption analysis results revealed that the diffusible hydrogen content decreased with an increasing tempering temperature. The hydrogen embrittlement resistance increased with decreasing the fraction of the diffusible hydrogen because the hydrogen embrittlement was mainly governed by the amount of diffusible hydrogen.

## Figures and Tables

**Table 1:** Microstructure characteristics of the SCM440 steels tempered at different temperatures.

Steel	Grain Boundary Fraction ^(1)^ (%)		Geometrically Necessary Dislocation (GND) Density ^(1)^ (10^14^/m^2^)	Dislocation Density ^(2)^ (10^14^/m^2^)
	Low-Angle Grain Boundary (<15°)	High-Angle Grain Boundary (>15°)		
As-quenched	24.8	75.2	4.59	4.81
Tempered at 300 °C	21.3	78.7	4.61	5.24
Tempered at 450 °C	21.3	78.7	4.29	2.60
Tempered at 600 °C	19.4	80.6	3.96	1.03

^(1)^ Measured by EBSD. ^(2)^ Calculated through Williamson–Hall plot method.

**Table 2:** Tensile properties and hydrogen embrittlement resistance of the SCM440 steels tempered at different temperatures.

Steel		Tensile Properties			Hydrogen Embrittlement Resistance	
		Yield Strength (MPa)	Tensile Strength (MPa)	Total Elongation (%)	Relative Reduction Area (RRA)	Relative Elongation (RE)
As-quenched	Non-charged	1421 ± 6	2190 ± 6	6.5 ± 0.4	0.03	0.01
	H-charged	111 ± 17	111 ± 12	0.08 ± 0.0		
Tempered at 300 °C	Non-charged	1379 ± 11	1857 ± 14	7.3 ± 0.5	0.05	0.01
	H-charged	180 ± 7	180 ± 8	0.07 ± 0.0		
Tempered at 450 °C	Non-charged	1204 ± 5	1422 ± 12	8.3 ± 0.1	0.09	0.04
	H-charged	628 ± 4	628 ± 8	0.3 ± 0.0		
Tempered at 600 °C	Non-charged	893 ± 5	1021 ± 7	12.4 ± 0.2	0.69	0.71
	H-charged	894 ± 6	1000 ± 6	8.5 ± 0.1		

## Data Availability

Data sharing not applicable.

## References

[1] Venezuela J., Liu Q., Zhang M., Zhou Q., Atrens A. (2016). A review of hydrogen embrittlement of martensitic advanced high-strength steels. Corros. Rev..

[2] Kim J.S., Lee Y.H., Lee D.L., Park K.T., Lee C.S. (2009). Microstructural influences on hydrogen delayed fracture of high strength steels. Mater. Sci. Eng. A.

[3] Hirata K., Iikubo S., Koyama M., Tsuzaki K., Ohtani H. (2018). First-Principles Study on Hydrogen Diffusivity in BCC, FCC, and HCP Iron. Metall. Mater. Trans. A.

[4] Takai K., Watanuki R. (2003). Hydrogen in Trapping States Innocuous to Environmental Degradation of High-strength Steels. ISIJ Int..

[5] Louthan M.R., Caskey G.R., Donovan J.A., Rawl D.E. (1972). Hydrogen embrittlement of metals. Mater. Sci. Eng. A.

[6] Koyama M., Akiyama E., Lee Y.K., Raabe D., Tsuzaki K. (2017). Overview of hydrogen embrittlement in high-Mn steels. Int. J. Hydrogen Energy.

[7] Pfeil L.B. (1926). The effect of occluded hydrogen on the tensile strength of iron. Proc. R Soc..

[8] Beachem C.D. (1972). A new model for hydrogen-assisted cracking (hydrogen “embrittlement”). Metall. Trans..

[9] Nagumo M. (2001). Function of Hydrogen in Embrittlement of High-strength Steels. ISIJ Int..

[10] Golovanenko S.A., Zikeev V.N., Serebryanaya E.B., Popova L.V. (1978). The influence of alloy elements and structure on the resistance of constructional steels to hydrogen embrittlement. Met. Term Obrab Met..

[11] Yoo J., Jo M.C., Kim S., Oh J., Bian J., Sohn S.S., Lee S. (2020). Effects of Ti alloying on resistance to hydrogen embrittlement in (Nb+Mo)-alloyed ultra-high-strength hot-stamping steels. Mater. Sci. Eng. A.

[12] Zhu X., Li W., Hsu T.Y., Zhou S., Wang L., Jin X. (2015). Improved resistance to hydrogen embrittlement in a high-strength steel by quenching–partitioning–tempering treatment. Scr. Mater..

[13] Nagao A., Hayashi K., Oi K., Mitao S. (2012). Effect of Uniform Distribution of Fine Cementite on Hydrogen Embrittlement of Low Carbon Martensitic Steel Plates. ISIJ Int..

[14] Lee J., Lee T., Mun D.J., Bae C.M., Lee C.S. (2019). Comparative study on the effects of Cr, V, and Mo carbides for hydrogen-embrittlement resistance of tempered martensitic steel. Sci. Rep..

[15] Peral L.B., Zafra A., Blason S., Rodriguez C., Belzunce J. (2019). Effect of hydrogen on the fatigue crack growth rate of quenched and tempered CrMo and CrMoV steels. Int. J. Fatigue.

[16] Zhang C., Liu Y., Jiang C., Xiao J. (2011). Effects of Niobium and Vanadium on Hydrogen-Induced Delayed Fracture in High Strength Spring Steel. J. Iron Steel Res. Int..

[17] Zafra A., Peral L.B., Belzunce J., Rodríguez C. (2019). Effects of hydrogen on the fracture toughness of 42CrMo4 steel quenched and tempered at different temperatures. Int. J. Press. Vessels Pip..

[18] Kang H.J., Yoo J.S., Park J.T., Ahn S.T., Kang N., Cho K.M. (2012). Effect of nano-carbide formation on hydrogen-delayed fracture for quenching and tempering steels during high-frequency induction heat treatment. Mater. Sci. Eng. A.

[19] Shi X., Yan W., Wang W., Shan Y., Yang K. (2016). Novel Cu-bearing high-strength pipeline steels with excellent resistance to hydrogen-induced cracking. Mater. Des..

[20] Kimura Y., Sakai Y., Hara T., Belyakov A., Tsuzaki K. (2003). Hydrogen induced delayed fracture of ultrafine grained 0.6% O steel with dispersed oxide particles. Scr. Mater..

[21] (2008). ASTM Standards. Standard Test Methods for Tension Testing of Metallic Materials.

[22] Saastamoinen A., Kaijalainen A., Heikkala J., Porter D., Suikkanen P. (2018). The effect of tempering temperature on microstructure, mechanical properties and bendability of direct-quenched low-alloy strip steel. Mater. Sci. Eng. A.

[23] Williamson G.K., Hall W.H. (1953). X-ray line broadening from filed aluminium and wolfram. Acta Metall..

[24] Langford J.I., Wilson J.C. (1978). Scherrer after sixty years: A survey and some new results in the determination of crystallite size. J. Appl. Crystallogr..

[25] Williamson G.K., Smallman R.E. (1956). Dislocation densities in some annealed and cold-worked metals from measurements on the X-ray debye-scherrer spectrum. Philos. Mag. A.

[26] Gao H., Huang Y., Nix W.D., Hutchinson J.W. (1999). Mechanism-based strain gradient plasticity—I. Theory. J. Mech. Phys. Solids.

[27] Zhang Y., Li C., Han L., Gu J. (2021). Effect of Tempering Temperature on Microstructure Evolution and Hardness of 9Cr1. 5Mo1CoB(FB2) Steel. Steel Res. Int..

[28] Badji R., Chauveau T., Bacroix B. (2013). Texture, misorientation and mechanical anisotropy in a deformed dual phase stainless steel weld joint. Mater. Sci. Eng. A.

[29] Masoumi M., Silva C.C., Abreu H.F.G. (2016). Effect of crystallographic orientations on the hydrogen-induced cracking resistance improvement of API 5L X70 pipeline steel under various thermomechanical processing. Corros. Sci..

[30] Oudriss A., Creus J., Bouhattate J., Conforto E., Berziou C., Savall C., Feaugas X. (2012). Grain size and grain-boundary effects on diffusion and trapping of hydrogen in pure nickel. Acta Mater..

[31] Mine Y., Tachibana K., Horita Z. (2011). Grain-boundary diffusion and precipitate trapping of hydrogen in ultrafine-grained austenitic stainless steels processed by high-pressure torsion. Mater. Sci. Eng. A.

[32] Takano N. (2006). Hydrogen diffusion and embrittlement in 7075 aluminum alloy. Mater. Sci. Eng. A.

[33] Mine Y., Tsumagari T., Horita Z. (2010). Hydrogen trapping on lattice defects produced by high-pressure torsion in Fe—0.01 mass% C alloy. Scr. Mater..

[34] Xu H., Xia X., Hua L., Sun Y., Dai Y. (2012). Evaluation of hydrogen embrittlement susceptibility of temper embrittled 2.25Cr-1Mo steel by SSRT method. Eng. Fail. Anal..

[35] Dutta R.K., Petrov R.H., Delhez R., Hermans M.J.M., Richardson I.M., Bottger A.J. (2013). The effect of tensile deformation by in situ ultrasonic treatment on the microstructure of low-carbon steel. Acta Mater..

[36] Lin Y.T., Yi H.L., Chang Z.Y., Lin H.C., Yen H.W. (2021). Role of vanadium carbide in hydrogen embrittlement of press-hardened steels: Strategy from 1500 to 2000 MPa. Front. Mater..

[37] Wang L., Cheng X.Y., Peng H., Zhao P.W., Cai Z.X. (2021). Effect of tempering temperature on hydrogen embrittlement in V-containing low alloy high strength steel. Mater. Lett..

[38] Rehrl J., Mraczek K., Pichler A., Werner E. (2014). Mechanical properties and fracture behavior of hydrogen charged AHSS/UHSS grades at high- and low strain rate tests. Mater. Sci. Eng. A.

[39] Lin Y.C., Chen D., Chiang M.H., Cheng G.J., Lin H.C., Yen H.W. (2019). Response of Hydrogen Desorption and Hydrogen Embrittlement to Precipitation of Nanometer-Sized Copper in Tempered Martensitic Low-Carbon Steel. JOM.

[40] Wei F.G., Tsuzaki K. (2005). Response of hydrogen trapping capability to microstructural change in tempered Fe—0.2C martensite. Scr. Mater..

[41] Doshida T., Takai K. (2014). Dependence of hydrogen-induced lattice defects and hydrogen embrittlement of cold-drawn pearlitic steels on hydrogen trap state, temperature, strain rate and hydrogen content. Acta Mater..

[42] Depover T., Verbeken K. (2016). The effect of TiC on the hydrogen induced ductility loss and trapping behavior of Fe-C-Ti alloys. Corros. Sci..

